# Connecting Artificial Brains to Robots in a Comprehensive Simulation Framework: The Neurorobotics Platform

**DOI:** 10.3389/fnbot.2017.00002

**Published:** 2017-01-25

**Authors:** Egidio Falotico, Lorenzo Vannucci, Alessandro Ambrosano, Ugo Albanese, Stefan Ulbrich, Juan Camilo Vasquez Tieck, Georg Hinkel, Jacques Kaiser, Igor Peric, Oliver Denninger, Nino Cauli, Murat Kirtay, Arne Roennau, Gudrun Klinker, Axel Von Arnim, Luc Guyot, Daniel Peppicelli, Pablo Martínez-Cañada, Eduardo Ros, Patrick Maier, Sandro Weber, Manuel Huber, David Plecher, Florian Röhrbein, Stefan Deser, Alina Roitberg, Patrick van der Smagt, Rüdiger Dillman, Paul Levi, Cecilia Laschi, Alois C. Knoll, Marc-Oliver Gewaltig

**Affiliations:** ^1^The BioRobotics Institute, Scuola Superiore Sant’Anna, Pontedera, Italy; ^2^Department of Intelligent Systems and Production Engineering (ISPE – IDS/TKS), FZI Research Center for Information Technology, Karlsruhe, Germany; ^3^Department of Software Engineering (SE), FZI Research Center for Information Technology, Karlsruhe, Germany; ^4^Computer and Robot Vision Laboratory, Instituto de Sistemas e Robotica, Instituto Superior Tecnico, Lisbon, Portugal; ^5^Department of Informatics, Technical University of Munich, Garching, Germany; ^6^Fortiss GmbH, Munich, Germany; ^7^Blue Brain Project (BBP), École polytechnique fédérale de Lausanne (EPFL), Genève, Switzerland; ^8^Department of Computer Architecture and Technology, CITIC, University of Granada, Granada, Spain

**Keywords:** neurorobotics, robot simulation, brain simulation, software architectures, robot programming, web technologies

## Abstract

Combined efforts in the fields of neuroscience, computer science, and biology allowed to design biologically realistic models of the brain based on spiking neural networks. For a proper validation of these models, an embodiment in a dynamic and rich sensory environment, where the model is exposed to a realistic sensory-motor task, is needed. Due to the complexity of these brain models that, at the current stage, cannot deal with real-time constraints, it is not possible to embed them into a real-world task. Rather, the embodiment has to be simulated as well. While adequate tools exist to simulate either complex neural networks or robots and their environments, there is so far no tool that allows to easily establish a communication between brain and body models. The Neurorobotics Platform is a new web-based environment that aims to fill this gap by offering scientists and technology developers a software infrastructure allowing them to connect brain models to detailed simulations of robot bodies and environments and to use the resulting neurorobotic systems for *in silico* experimentation. In order to simplify the workflow and reduce the level of the required programming skills, the platform provides editors for the specification of experimental sequences and conditions, environments, robots, and brain–body connectors. In addition to that, a variety of existing robots and environments are provided. This work presents the architecture of the first release of the Neurorobotics Platform developed in *subproject 10 “Neurorobotics”* of the Human Brain Project (HBP).^1^ At the current state, the Neurorobotics Platform allows researchers to design and run basic experiments in neurorobotics using simulated robots and simulated environments linked to simplified versions of brain models. We illustrate the capabilities of the platform with three example experiments: a Braitenberg task implemented on a mobile robot, a sensory-motor learning task based on a robotic controller, and a visual tracking embedding a retina model on the iCub humanoid robot. These use-cases allow to assess the applicability of the Neurorobotics Platform for robotic tasks as well as in neuroscientific experiments.

## 1 Introduction

Developing neuro-inspired computing paradigms that mimic nervous system functions is a well-established field of research that fosters our understanding of the human brain. The brain is a complex structure, and designing models that can mimic such a structure is particularly difficult. Modeling brain function requires understanding how each subsystem (sensory, motor, emotional, etc.) works, how these subsystems interact with each other, and, as a whole, how they can generate complex behaviors in the interaction with the environment. Moreover, it is well known that during development the brain is molded by experience and the environment (Benefiel and Greenough, 1998; Briones et al., 2004). Thus, studying and validating models of brain function requires a proper embodiment of the brain model as well as a dynamic and rich sensory environment in which the robot–brain ensemble can be embedded and then be exposed to a realistic sensory-motor task. Since advanced brain models are too complex to be simulated in real time, the researcher is faced with a dilemma. Either the brain model is simplified until it can be simulated in real time. In this case, the brain model can be embedded in a physical robot, operating in the real world, but the complexity of the brain models that can be studied is highly limited. Or the complexity of the brain model is maintained. In this case, there are no limits on the brain models; however, it is now no longer possible to embed the brain into a real-world task. Rather, the embodiment has to be simulated as well.

While adequate tools exist to simulate either complex neural network models (Gewaltig and Diesmann, 2007) or robots and their environments (Koenig and Howard, 2004), there is so far no tool that allows researchers to easily connect realistic brain models to a robot and embed it in a sensory-rich environment model.

Such a tool would require the capability of orchestrating and synchronizing both simulations as well as managing the exchange of data between them. The goal of such simulations is to study and quantify the behavior of models of the brain. As a consequence, we do not only need a complex, realistic experimental environment but we also need a controllable and measurable setup where stimuli can be generated and responses can be measured. In fact, real environment complexity and parameters are intrinsically difficult or even impossible to control. In addition, models of brain functions, designed to properly reproduce brain activity at different levels could not be executed in real time due to complex neuron dynamics and the size of the network (Kunkel et al., 2014). This is the reason why we propose to use a digital simulator implementing realistic scenarios. The main restriction we propose is to have a simulator that could run at a “slower” time (limited by the computation time required by the brain simulation) and also that the time can be sampled in discrete intervals without compromising the simulation quality.

The idea behind this approach is providing a tool chain, which grants researchers’ access to simulation control as well as state-of-the-art tools such as models of robot and brain and methods to connect them in a proper way (i.e., connecting spiking neural networks to robotic sensors and actuators). A first approach used to connect spiking neural networks and robots has been presented by Gamez et al. (2012). iSpike is a C++ library that provides an interface between spiking neural network simulators and the iCub humanoid robot. It uses a biologically inspired approach to convert the robots’ sensory information into spikes that are passed to the neural network simulator, and it decodes output spikes from the network into motor signals that are sent to control the robot. Another communication interface named CLONES (Voegtlin, 2011) between a neural simulator [BRIAN (Goodman and Brette, 2008)] and SOFA, a physics engine for biomedical applications (Allard et al., 2007), has been developed using shared memory and semaphores. The most similar system to iSpike and CLONES is the interface that was created for the CRONOS and SIMNOS robots (Gamez et al., 2006) which encoded visual and proprioceptive data from the robots into spikes that were passed to a spiking neural network simulated in SpikeStream. Spiking motor output from the network was transformed back into real values that were used to control the robots. This system was used to develop a spiking neural network that controlled the eye movements of SIMNOS, learnt associations between motor output and visual input, and used models of imagination and emotion to avoid negative stimuli. All these systems provide an interface toward specific robotic platforms able to deal with spiking/digital inputs and convert them appropriately. Together with robotic platform restrictions, they do not provide a framework for the conversion, allowing the user to write his own transfer function. A more generic system which permits dealing with simulated robotic platforms is AnimatLab (Cofer et al., 2010b). AnimatLab currently has two different neural models that can be used. One is an abstract firing rate neuron model, and the other is a more realistic conductance-based integrate-and-fire spiking neural model. It is also possible to add new neural and biomechanical models as plug-in modules. There are several different joint types and a host of different body types that can be used. Although AnimatLab does not provide a comprehensive set of neurons and learning models, some behavior implementation based on this tool is available such as locust jumping (Cofer et al., 2010a) or dominant and subordinate crayfish (Issa et al., 2012). Despite some of the mentioned tools represents a good attempt to connect artificial brains to robots, these are not very common in the robotic and neuroscientific communities likely due to the limitations we have underlined (robotic platform restrictions, lack of a framework for conversions). For our framework, we decided to rely on widely used simulators for the brain models as well as for robots and environments. This strategic choice should allow to easily attract users of these platforms. We embedded these simulators in a comprehensive framework that allows the user to design and run neurorobotic experiments. In line with our approach, Weidel et al. (2015, 2016) proposed to couple the widely used neural simulation tool NEST (Gewaltig and Diesmann, 2007) with the robot simulator Gazebo (Koenig and Howard, 2004), using the MUSIC middleware (Djurfeldt et al., 2010).

Here, we describe the first release of the HBP Neurorobotics Platform, which offers scientists and technology developers a set of tools, allowing them to connect brain models to detailed simulations of robot bodies and environments and to use the resulting neurorobotic systems in *in silico* experiments and technology development. The Neurorobotics Platform (NRP) also provides a comprehensive development framework including editors for creating experiments, environments, and brain and robot models. These tools are accessible via the web allowing them to use the platform without tedious installation of software packages. Moreover, through the web, researchers can collaborate and share their models and experiments with their colleagues or with the scientific community.

Although the capabilities to model virtual robots and environments already exist as confirmed by the mentioned works, and although various labs have created closed-loop setups with simple brain models (Ros et al., 2006; Denoyelle et al., 2014), this platform is the first to allow the coupling of robots and detailed models of the brain. This makes it possible to perform experiments exploring the link between low-level brain circuitry and high-level function.

The aim of this platform is twofold: from one side, the platform can be used to test neuroscientific models of brain areas, or even reconstruction of these areas based on neurophysiological data; on the other side, roboticists can take advantage of such a platform to develop more biologically inspired control architectures. The physical and neural simulation are properly synchronized, and they exchange data through transfer functions that translate sensory information coming from the robot (camera image, encoders, etc.) into input for the brain (current and spikes) from one side and the network output into motor commands from the other. Additionally, the platform also provides a web interface, so that it can be easily accessed and used from a broader user base. From this web interface, the user can also access the editors that are used to construct experiments from scratch and run the experiments without any software installation, benefiting from the available computing and storage platforms that have been made available to support the NRP. Therefore, the NRP provides a complete framework for neurorobotics experiment design and simulation. One of the pillars of the NRP development is the reuse and extension of existing software, thus many components were implemented using suitably chosen existing software.

## 2 Platform Requirements

### 2.1 Functional Requirements

In order to obtain the *functional requirements* for the NRP, we first determined which features are needed for the creation of a neurorobotic experiment. In that, we followed software engineering concepts and terminologies to itemize platform features as requirements (IEEE, 1998). These features can be divided into two categories: *design* features and *simulation* features, each with its own functional requirements.

During the *design* of a neurorobotic experiment, the user should be able to define all of its properties, and this includes

- the design of a suitable *Robot* model, by defining both kinematic and dynamic properties as well as the appearance, either from scratch or from preexisting models;
- the possibility to create a rich *Environment* models in which the robot can operate, by using a library of objects;
- the design of a *Brain* model, either from scratch or by selecting an existing model, that will be coupled to the robot;
- *Brain–Body Integration*, in order to specify how the brain model and the robot should be coupled in terms of sensory and motor data exchange to create a *Neurobot*;
- the capability to change dynamic properties of the *Experiment* itself, like defining events that can be triggered during the simulation and appropriate response behaviors.

When all properties are defined, the *simulation* can start. During the execution, the NRP should provide

- World maintenance and synchronization mechanisms in order to not only simulate both the physics and neural models but also to synchronize the two simulations and exchange data between them, providing a closed-loop control mechanism, as defined in the design phase. It must be possible to start, pause, stop, and reset the simulation. The simulation should react to the triggered events previously defined;
- a proper *Interactive visualization* of the running simulation, comprising a GUI and utilities to see details of the simulation like brain activity or robot parameters. Moreover, the user should be able to *live edit* and interact with the simulation once it is started, using the same design features described above.

**Figure 1:**
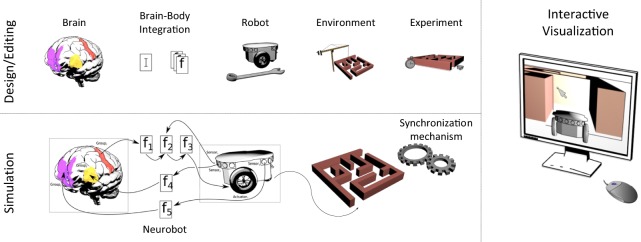
**Functional overview of the Neurorobotics Platform**. Using the design/editing features of the platform, the user is able to create a neurorobotic experiment comprising of a brain model integrated with a robotic body (Neurobot) that interacts in a dynamic environment. The experiment is then simulated by a synchronized neural-physics simulation, and the results can be displayed in an interactive fashion.

A complete list of functional requirements can be found in Appendix A, while an overview of the platform functionalities is shown in Figure 1.

### 2.2 Non-functional Requirements

Several non-functional requirements were also defined:

- *usability and user experience*—the platform should be easily accessible to a wide range of users that possibly have no experience in either the neuroscientific or robotic fields. This should be achieved by a user-centric design with intuitive tools and a consistent user experience. Moreover, the platform should also provide an additional user level in order for expert users to have more detailed design capabilities.
- *open source*—the NRP should rely on existing building blocks, and in particular on open source ones, as the platform has to be released to a wide audience.
- *interoperability*—each software component that allows to save or load data should use, wherever possible, well-known data formats.
- *software quality*—in order to ensure software quality, the development of the platform should follow software engineering practices such as keeping a task tracking system, using version control with code review and continuous builds, and employing standard software development methodologies.

### 2.3 Integration with Other HBP Platforms

The NRP is one of six platforms developed in the Human Brain Project. In addition to the Neurorobotics Platform, the HBP develops a *Neuroinformatics Platform*, a *Brain Simulation Platform*, a *High Performance and Data Analytics Platform*, a *Neuromorphic Computing Platform*, and a *Medical Informatics Platform*. Most of these offer their services through the web and are built on top of a common set of APIs and services, called *HBP Collaboratory Portal*. It provides the following services:

- Authentication, access rights, and user profiles. The users are provided with a Single Sign-On mechanism so they can use the same credentials to access every HBP platform.
- Document repository. The users have access to a document repository in which they can store and manage their projects. It supports one of NRP’s requirements, namely, the possibility for the users to share their models (brain, connections, environment, robots, or experiments) with team members.
- Collaboratory API. A web-based application with associated libraries allowing every platform’s web interface to have the same look and feel, and to be implemented as a plugin within the Collaboratory Portal.

All the HBP platform should provide some level of integration among each other. For this reason, short-term future development plans include the integration of the Neurorobotics Platform with the Brain Simulation Platform, the Neuromorphic Computing Platform, while in the long-term integration with the High Performance Computing and Analytics Platform will also be provided.

The Brain Simulation Platform aims at providing scientists with tools to reconstruct and simulate scaffold models of brain and brain tissue using data from within and outside the HBP. The Brain Simulation Platform will be integrated with the NRP for simulating brain models at various detail levels. Moreover, alongside the Brain Simulation Platform, scaffold brain models will be gathered and they will be available for usage in the platform.

The Neuromorphic Computing Platform provides remote access to large-scale neuromorphic computing systems built in custom hardware. Compared to traditional HPC resources, the neuromorphic systems offer higher speed (real time or accelerated simulation time) and lower energy consumption. Thus, the integration of the platform will provide an alternative neural simulation backend more suitable for simulations that require a high computational burden, such as in experiments involving plasticity and learning.

## 3 Software Architecture

**Figure 2:**
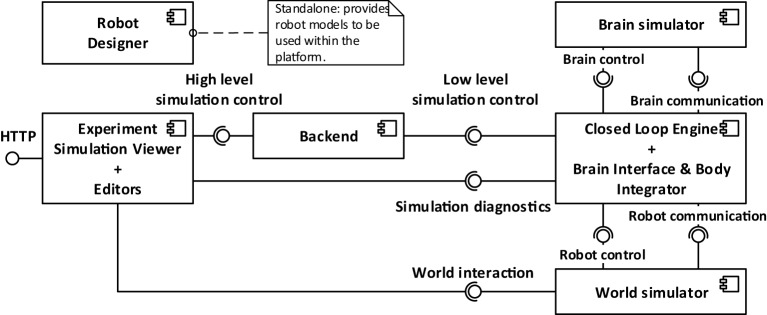
**Architectural overview of the platform**. From left to right, three layers can be distinguished: the user interface (Experiment Simulation Viewer), the services connecting the user interface to the simulations (implemented in the Backend), and the internal computations, comprising the two simulations and the synchronization between them.

The Neurorobotics Platform is based on a three-layer architecture, shown in Figure 2.

The layers, starting from the one furthest from the user, are the following:

1. the software components simulating the neurorobotics experiment;
2. the *REST server* or *Backend*;
3. the *Experiment Simulation Viewer (ESV)*, a graphical user interface, and the Robot Designer, a standalone application for the design of physical models.

The first layer comprises all the software components that are needed to simulate a neurorobotics experiment. The *World Simulation Engine (WSE)* is responsible for simulating robots and their environment. The *Brain Simulator* is responsible to simulate the neural network that controls the robot. The *Closed Loop Engine (CLE)* implements the unique logic of each experiment and orchestrates the interaction between the two simulators and the ESV.

The second layer contains the *REST server*, also referred to as *Backend*, which receives requests from the ESV and forwards them to the appropriate components, which implements the requested service, mainly through ROS. The REST server thus acts as a relay between the graphical user interface (the frontend), and the various simulation engines needed for the neurorobotics experiment. For practical reasons, the services provided by the REST server are tightly coupled with the high-level functionality shown in the ESV GUI. Thus any graphical control interacting with the REST server has a corresponding service. Actions that change the state of the simulations, such starting, stopping, or pausing a simulation, are implemented as a single parametric service.

The ESV is the web-based graphical user interface to all neurorobotics experiments. Using the ESV, the user can control and visualize neurorobotics experiments. The ESV also provides a number of *editors* to configure the experiment protocol as well as the parts of the experiment such as the environment, the brain model, and the connection between brain and robots (*Brain Interface and Body Integrator*). The Robot Designer is a tool that was developed to allow the process of designing robot models that can be included in simulation setups executable on the NRP. This tool is developed as a plugin for the 3D modeling tool Blender 3D.^2^

### 3.1 Brain Simulator

The goal of the *Brain Simulator* is to simulate a brain circuit, implemented with a spiking neural network (SNN).

Several simulators for SNNs exist, with different levels of detail, ranging from more abstract point neuron simulations, which consider neural networks as directed graphs, to the morphologically accurate ones where the properties of axons and dendrites are taken into account.

Inside the NRP, the simulator currently supported is NEST (Gewaltig and Diesmann, 2007), a point neuron simulator with the capability of running on high-performance computing platforms, that is also one of the simulation backends of the Brain Simulation Platform. NEST is supported through the use of the PyNN abstraction layer (Davison et al., 2008) that provides the same interface for different simulators and also for neuromorphic processing units, i.e., dedicated hardware for the simulation of SNN such as SpiNNaker (Khan et al., 2008), provided by the Neuromorphic Computing Platform. Both NEST and PyNN provide convenient mechanisms to design neural networks. Furthermore, they are among the most used tools in the neuroscientific community. On the other hand, the only APIs they provide are written in Python, which heavily constraints the choice of the language to use for interacting with them.

### 3.2 World Simulator

In order to have realistic experiments, the accurate brain simulation must be coupled with a detailed physics simulation. The *World Simulator* component aims at delivering a realistic simulation for both the robot and the environment in which the robot interacts.

Gazebo was chosen as the physics simulator. It offers a multi-robot environment with an accurate simulation of the dynamics, in particular gravity, contact forces, and friction. This dynamic simulation can be computed with different supported software libraries like ODE (Drumwright, 2010) and Bullet (Coumans et al., 2013).

Any communication with the simulated robot and control of the simulation itself is done through the Robot Operating System (ROS) (Quigley et al., 2009), which is natively integrated with Gazebo.

ROS is a widely used middleware in the robotics community and provides C++ and Python APIs to the user.

### 3.3 Brain Interface and Body Integrator

The *Brain Interface and Body Integrator* (BIBI) plays a crucial role in the NRP, as it is the component that implements the connection between the robot and brain simulations. The main feature of the BIBI is the *Transfer Function* framework. A Transfer Function (TF) is a function that translates the output of one simulation into a suitable input for the other. Thus, we can identify two main types of transfer functions: the *Robot to Neuron* TFs translate signals coming from robot parts such as sensor readings and camera images into neuron signals such as spikes, firing rates, or electric currents; the *Neuron to Robot* TFs convert neural signals from individual neurons or groups of neurons into control signals for robot motors. Thus, these two kinds of transfer functions close the action–perception loop by filling the gaps between the neural controller and the robot.

The TFs also extend beyond the previously described two types. For example, the robot–brain–robot loop can be short-circuited in order to bypass the brain simulation and use only a classical robotic controller, thus resulting in a *Robot to Robot* TF. This allows the comparison between a classical and a neural implementation of a robotic controller with the same setup, by simply switching from a transfer function to another. Moreover, the data coming from both simulations can be sent out of the loop (to a monitoring module) where it can be live plotted, elaborated, stored, or exported for data analysis with external tools (*Robot to Monitor* and *Neuron to Monitor* TFs).

In order to provide a proper abstraction layer toward the simulators, generic interfaces are provided, which are then implemented by specific adapters. From the robot simulator side, the interface is modeled following the publish–subscribe design pattern (Gamma et al., 1995), where, from one side, sensory information is expected to be published by the robotic simulator and the *Robot to Neuron* TF subscribes to the subject, receiving the data, while on the other side the *Neuron to Robot* TF publishes motor commands and the simulator is expected to subscribe and execute them. This pattern is used by many robotics middlewares such as ROS and YARP (Metta et al., 2006), thus there is minimal work required in order to implement the adapters in such cases. In the current implementation of the NRP, ROS Topic adapters have been implemented. From the brain simulation side, the TFs provide stimuli and measurements by using *Devices*. Devices are abstract entities that have to be connected to the neural network, either to a single neuron or to a neuron population. Among such entities, there are spike generators and current generators (for the input side), and spike recorders, population rates recorders, and leaky integrators (for the output side). In the current implementation, devices are implemented as wrappers around PyNN objects instances, providing general interfaces toward different neural simulators.

The TF framework is implemented using the Python programming language, where the business logic of each TF resides in a function definition. A library of commonly used transfer functions, including common image processing primitives and simple translational models for motor command generation, is provided alongside with the framework. Information about the TF connections is specified via a custom Domain Specific Language (DSL) implemented with Python decorators that specify the type of transfer function, the device types, and the neuron which they are connected to, and the topics that the TF should subscribe to, or on which topic the TF should publish (Hinkel et al., 2015, 2017). An example of a transfer function implementation is displayed in Listing 1.

Listing 1 **An example of transfer function code, translating an image into spike rates.**

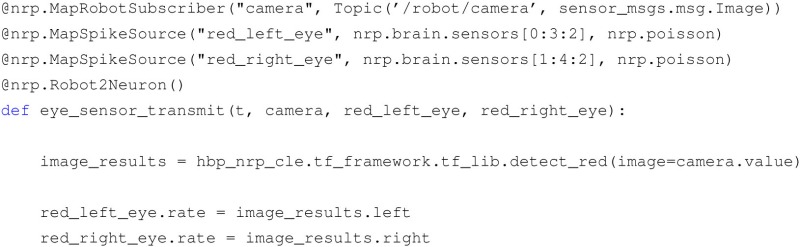

In this example, it can be seen that through the use of the decorators DSL several properties are specified, such as the type of TF (Robot to Neuron), the devices toward the brain simulation (spike generators firing with Poisson statistics attached to the neuron population) and the input coming from the robotic simulation (camera image published through a ROS topic). It can also be noticed that the actual business logic is implemented inside the function, and in particular, the image is processed with a color detection filter implemented as part the TF library provided alongside the platform.

The choice of Python for the TF framework was the most natural one, given the fact that both the chosen physics and neural simulators provide Python APIs. Consequently, the rest of the server side NRP components have been written in Python. In principle, this could raise performance issues when compared with languages like C++. We chose to avoid fine tuning of the performance of the developed components, as currently the bottlenecks of a simulation reside in the physics and neural simulators. This choice has also the advantage of simplifying considerably the development process.

Internally, the complete BIBI configuration, comprising the transfer functions, the robot model, and the brain model, is stored as an XML file. Each transfer function can be saved either as Python code in an XML tag or can be constructed from custom XML elements which are later parsed in order to generate the equivalent Python code. The second way of describing these functions is better suited for the automatic generation of such XML files, via graphical editors that could be used also by scientists with no experience in Python.

### 3.4 Closed Loop Engine

**Figure 3:**
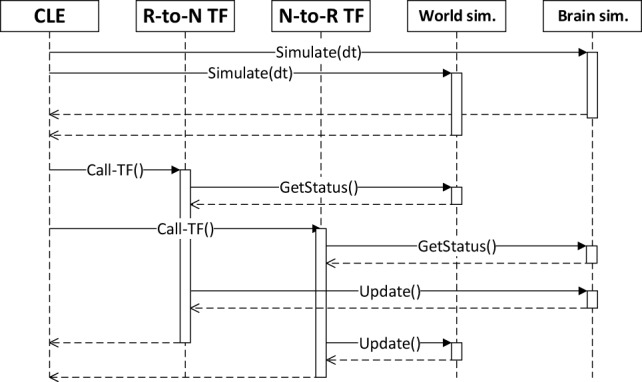
**Synchronization between the components of a simulation, as orchestrated by the CLE**. In a first phase, the two simulations are run in parallel. Afterward, each transfer function gathers data from simulations and computes the appropriate inputs for the next simulation step.

The *Closed Loop Engine* (CLE) is responsible for the control of the synchronization as well as for the data exchange among the simulations and the TFs. The purpose of the CLE is to guarantee that both simulations start and run for the same timestep, and also to run the TFs that will use the data collected at the end of the simulation steps. Figure 3 shows a sequence diagram of a typical execution of a timestep: after the physics and neural simulations have completed their execution in parallel, the TFs receive and process data from the simulations and produce an output which is the input for the execution of the next step. The idea behind the proposed synchronization mechanism is to let both simulations run for a fixed timestep, receiving and processing the output of the previous steps and yielding data that will be processed in the future steps by the concurrent simulation. In other words, data generated by one simulation in the current timestep cannot be processed by the other simulation until the next one. This can be read as the TFs introducing a delay of sensory perception and motion actuation greater than the simulation timestep.

We decided not to use MUSIC for the synchronization in this first release, even if it was shown to be working by Weidel et al. (2015, 2016), in order to ease the communication between brain and world simulations without introducing any middle layer. Moreover, relying on the already existing Python APIs for the communication with the two simulators had the effect of simplify the development process.

Besides orchestrating running simulations, the CLE is also responsible of spawning new ones, by creating new dedicated instances of the World Simulator and the Brain Simulator, and a new instance of the orchestrator between the two.

#### 3.4.1 Simulation Control

**Figure 4:**
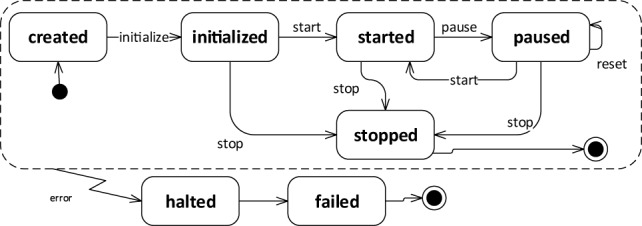
**Lifecycle of a simulation in the NRP**. During a normal cycle, the simulation will start from the *created* state, passing through *initialized* as soon as the resources are instantiated, then going through the *started* state once the execution is initiated, and finally in the *stopped* state. During the execution, the simulation can be *paused* at any time, while if any error occurs during the normal lifecycle the simulation is *halted*.

During its life cycle, each simulation transitions through several states, as depicted in Figure 4. At the beginning, a simulation is in state *created*, and it will switch to state *initialized* once the CLE is instantiated. Up to this point, no simulation steps have been performed yet. Once the simulation is *started*, the CLE will start the interleaving cycle that can be temporarily interrupted by pausing the simulation (*paused*) or preemptively terminated by stopping the simulation (*stopped*). If any error occurs during the execution or during the transitions between states, the simulation will pass automatically to the state *halted*. The *reset* transition can be considered parametrized, as it allows restoring to their initial status separate parts of the simulation singularly. Currently, the resettable parts in a simulation are the robot pose, the brain configuration, and the environment.

Thanks to the possibility of pausing and restarting the closed loop cycle during the simulation execution, it was possible to add features that modify simulation properties at runtime, without the need to restart the simulation from scratch. These features include support for transfer function adding, editing and removal, brain model, and environment editing. Using these features, it is possible to test different configurations of the simulation and immediately see the effects of them, without having to wait for a complete restart.

**Figure 5:**
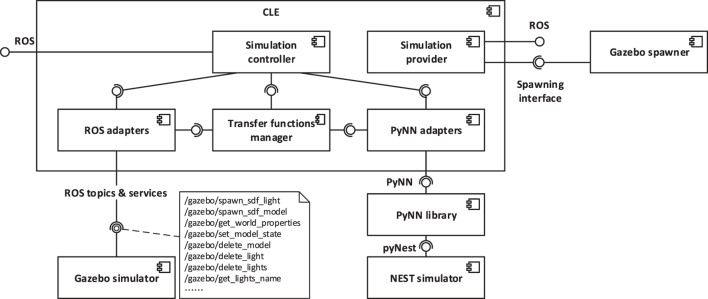
**Architectural overview of the CLE and of the communication layers**. The CLE orchestrates the two simulations and performs the data exchange through generic adapter interfaces. It also provides two interfaces, one for controlling an ongoing simulation and one for providing a new one, by instantiating a neural simulator and a physics simulator. In the current implementation, adapters for accessing Gazebo physics simulation via ROS and for accessing NEST neural simulation via PyNN are provided. In particular, robot data are accessed through ROS services and topics, and the physics simulation is controlled through ROS services.

From the point of view of the implementation, the timestep of the physics simulation is sent to Gazebo through a ROS service call, while the brain simulation is directly run for the desired timestep with a PyNN call, as it can be observed from the architecture depicted in Figure 5. ROS service calls and the PyNN calls are implemented through generic adapter interfaces and perform a client-server interaction. Hence, in principle, a CLE instance can interact with different simulators than the ones currently supported (Gazebo and NEST). This abstraction layer, besides providing the possibility to change with relative ease the underlying simulators, simplifies the update process of the simulators, by limiting the number of files that need to be changed in response to a possible API update.

#### 3.4.2 State Machines for Simulation Control

In real experiments, it is often the case that the environment changes in response to occurring events, generated by the behavior of the subject, by the experimenter or automatically generated (i.e., timed events). Thus, in order to reproduce this behavior, the possibility to generate events that can influence the environment was added to the platform. In particular, the user can interact with some objects without having to interrupt the simulation, like changing the brightness of lights or screen colors, and an event system is provided. The event system is implemented with a state machine that is programmable by the user. In the current implementation, support for timed events is provided, allowing the user to program changes in the environment that have to occur at specific points in time.

The event system is managed by the *State machines manager*, implemented using the SMACH state machine framework (Bohren and Cousins, 2010) that is already integrated into ROS. Using such a framework, it is possible to program timed events that directly call Gazebo services in order to modify the environment.

### 3.5 Backend

**Figure 6:**
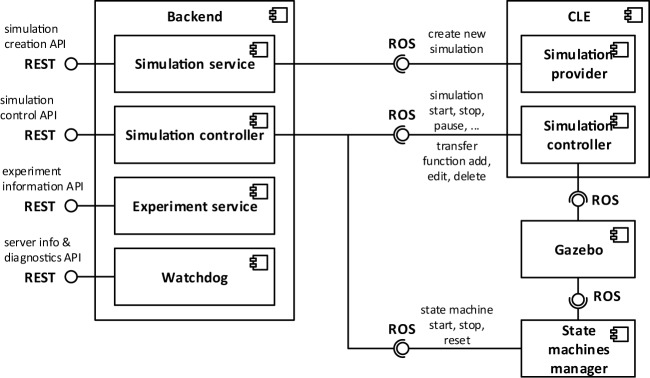
**Architectural overview of the Backend**. User inputs coming from the ESV are sent to the Backend via REST APIs. These requests are then dispatched to the CLE or to the State machine manager that will handle them by forwarding the appropriate commands to Gazebo.

The *Backend* is the component connecting the user interface to the core components of the platform, exposing a web server implementing RESTful APIs on the user interface end point and forwarding processed user requests via ROS on the other end point. This component is the first handler for user requests. In case they could not be completely managed within the backend, they are forwarded either to the CLE or to the State machines manager that will eventually complete the request processing, interacting, if necessary, with the simulators. An overview of the Backend architecture and of the interaction with other components is depicted in Figure 6.

Actions provided by the backend to the user interface (ESV) include experiment listing and manipulation, simulation listing, handling and creation, and gathering of backend diagnostic and information.

Every available experiment on the platform is identified by a name and a group of configuration files, including a preview image to be showed on the ESV and files representing environment, brain, state machines, and BIBI, where neural populations and transfer functions are stored. Experiment listings and manipulation APIs allow the user to list all the available experiments on the server as well as retrieving and customize singularly the configuration files of the experiment.

In the NRP setting, a simulation is considered as an instance of one of the available experiments. In order to create a new simulation, the user has to proceed in a different way depending on whether the NRP is accessed within or outside of the Collaboratory Portal. If users are accessing from the Collaboratory Portal, they are able to clone the configuration files related to one of the available experiments on the Collaboration storage they are using and instantiate that local copy of the experiment. The backend allows users to overwrite said configuration files as well as saving CSV recordings of simulation data directly on the storage. In case a user is not working from the Collaboratory Portal, they can instantiate an experiment choosing directly from the experiment list, and they can edit it without having to instantiate a local copy.

Once a simulation is created, the backend allows the user to retrieve and change its current state according to the simulation lifecycle depicted in Figure 4, by interfacing with the CLE. Other APIs provide functionalities for retrieving and editing at runtime the brain configuration, the state machines, and the transfer functions, delegating again the task to the CLE. Furthermore, information about the simulation metadata, brain populations, and environment configuration is available through dedicated APIs.

For diagnostic purposes, the backend provides some APIs for retrieving the errors which have occurred on the server as well as the version of the backend itself and the CLE.

### 3.6 Experiment Simulation Viewer

**Figure 7:**
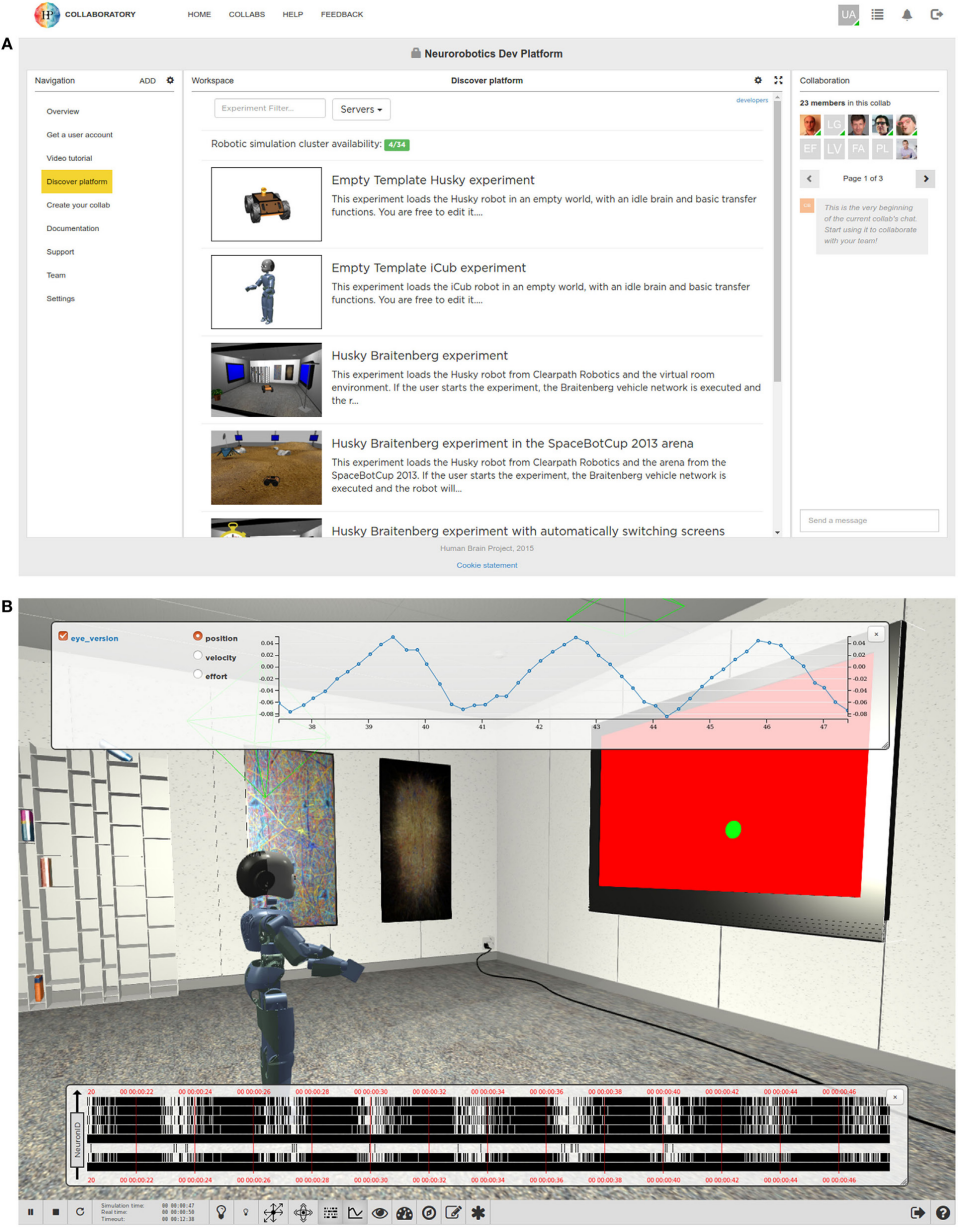
**The Experiment Simulation Viewer**. Through the Collaboratory Portal **(A)**, the user can choose between several predefined experiments or create his own experiment based on templates. Some of the features of the Collaboratory Portal such as a navigation pan and a group chat are shown. **(B)** The ESV Main View, where an experiment is being executed. The user interface at the bottom allows the user to interact with the simulation and displays information about simulation time. Some widgets, togglable from the user bar, allow the user to monitor brain activity in term of spike trains or joint values of the simulated robot.

The Experiment Simulation Viewer is the user interface to the NRP. It is implemented as a web-based application, developed using a modern web software stack exploiting established open-source software. The ESV is currently integrated in the Collaboratory Portal (see Figure 7A) using the Collaboratory APIs. By building it using standard web technologies, cross-platform support, also for mobile devices, is enabled. The downside of this choice is the added complexity of using translation layers, albeit lightweight ones, for the interaction with server-side components.

The ESV simulation interface embeds a 3D view that allows the user to see and navigate through the virtual environment, and a user bar for simulation control (e.g., for playing, pausing, resetting, or stopping the ongoing simulation). It also provides means for editing objects by altering their attributes and monitoring brain activity and the state of the embodiment, on a running simulation. Furthermore, the simulation interface hosts the tools that allow the user to design and edit an experiment, explained in depth in Section 3.6.3. Any modifications to the running simulation can be exported either on the user computer or saved on Collaboratory storage.

In the following sections, we start presenting the ESV user interface, its architecture, and then we continue describing the design tools.

#### 3.6.1 User Interface

Entering the ESV, the user is presented with a list of available experiments (see Figure 7A). For each experiment, the user can choose to launch a new simulation, or to join an already launched one as a spectator; it is also possible to launch an instance of an existing simulation while uploading a custom environment in which it will be executed, thus replacing the original one.

The user starting a simulation is called the owner of that simulation whereas any other user is called a watcher. The owner has full control over his simulation, being able to start, pause, stop, or reset the simulation and interact with the environment while it is running. Other features like monitoring or navigation into the scene are accessible to both owners and watchers.

Of particular interest are the monitoring features (Figure 7B). The Spike Monitor plots a spike train representation of the monitored neurons in real time. Monitored neurons must be specified by transfer functions, as described in Section 3.3.

The Joint Monitor plots a line chart representation of the joint properties of the robot in real time. For every joint selected, properties like position, velocity, and effort can be chosen.

The goal of these monitoring tools is to get live insights on how the simulation performs. Both spike data and joint data can also be saved in CSV format for further off-line analysis, see Section 3.6.3.3.

#### 3.6.2 Architecture

**Figure 8:**
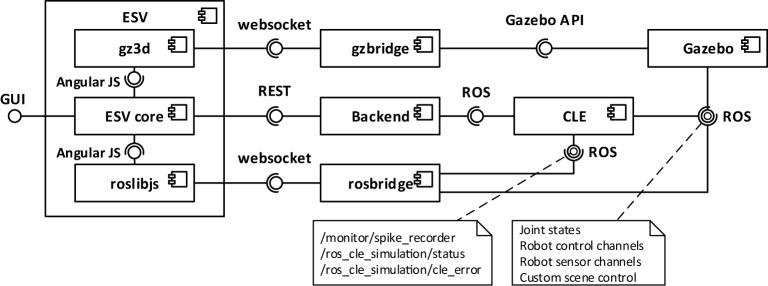
**Architectural overview of the ESV and its outgoing communications**. The ESV core interacts with the Backend via REST APIs to control the simulation. Two websocket channels bypass the Backend and allow the ESV to interact directly with Gazebo and the CLE. The gzbridge channel is used for gathering information about the scene rendering. The rosbridge channel is used for collecting information related to brain spikes and joint angles, which is shown on appropriate monitoring widgets in the GUI, and to get information about simulation status and possible errors.

In order to have a coherent user interface and experience throughout, all the tools developed in the Human Brain Project, including the NRP User interface are implemented as web applications. An architectural overview is shown in Figure 8.

The application framework of choice is AngularJS.^3^ AngularJS is a Model View Controller (MVC) Web Framework for developing single-page applications. Using AngularJS services, the interaction with the NRP Backend, which provides the API for the simulation control, is realized via standard REST calls.

The Rendering of the 3D view of the virtual environment is performed by Gzweb, Gazebo’s WebGL client. It comprises two main parts: gz3d and gzbridge, which are, respectively, responsible for visualization and for communicating with Gazebo’s backend server gzserver.

To enable the communications with the CLE and Gazebo via ROS, the ESV employs roslibjs, a JavaScript library. Roslibjs in turn interacts via WebSockets with rosbridge, a tool providing a JSON API to ROS functionality for non-ROS programs.

#### 3.6.3 Editors

In order to design the experiment to be simulated, the NRP provides the user with a complete array of tools. Thanks to these tools, it is possible to configure all the aspects of an experiment: *Environment, Transfer Functions, Brain*, and *Experiment Workflow*.

##### 3.6.3.1 Environment Editor

The purpose of the Environment Editor is to allow the user of the platform to set up the scene in which the simulation will run, either starting from scratch or editing one from an existing experiment.

The Environment Editor is seamlessly integrated into the ESV application: this dramatically shortens the time needed to prototype the experiment. Switching between simulation and editing the environment is a very fast process: the user can immediately simulate the interaction of the robot with the new environment and, if not satisfied, directly modify it again.

**Figure 9:**
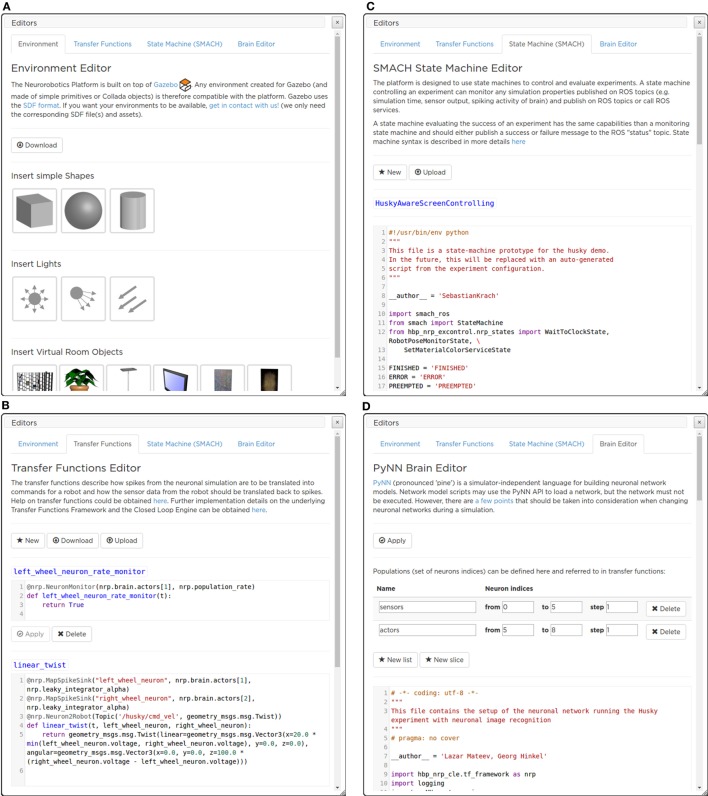
**The ESV editors’ menu panes**. With the Environment Editor **(A)** the user can add an object to the environment, choosing from a library of models. The Transfer Function editor **(B)** allows a live editing of the Transfer Functions, without the need for restarting the simulation. SMACH State Machine Editor **(C)** that currently implements the Experiment Workflow Editor, actions that have to be performed by the State machine manager can be defined. The brain model used in the simulation can be edited with the Brain Editor **(D)**.

**Figure 10:**
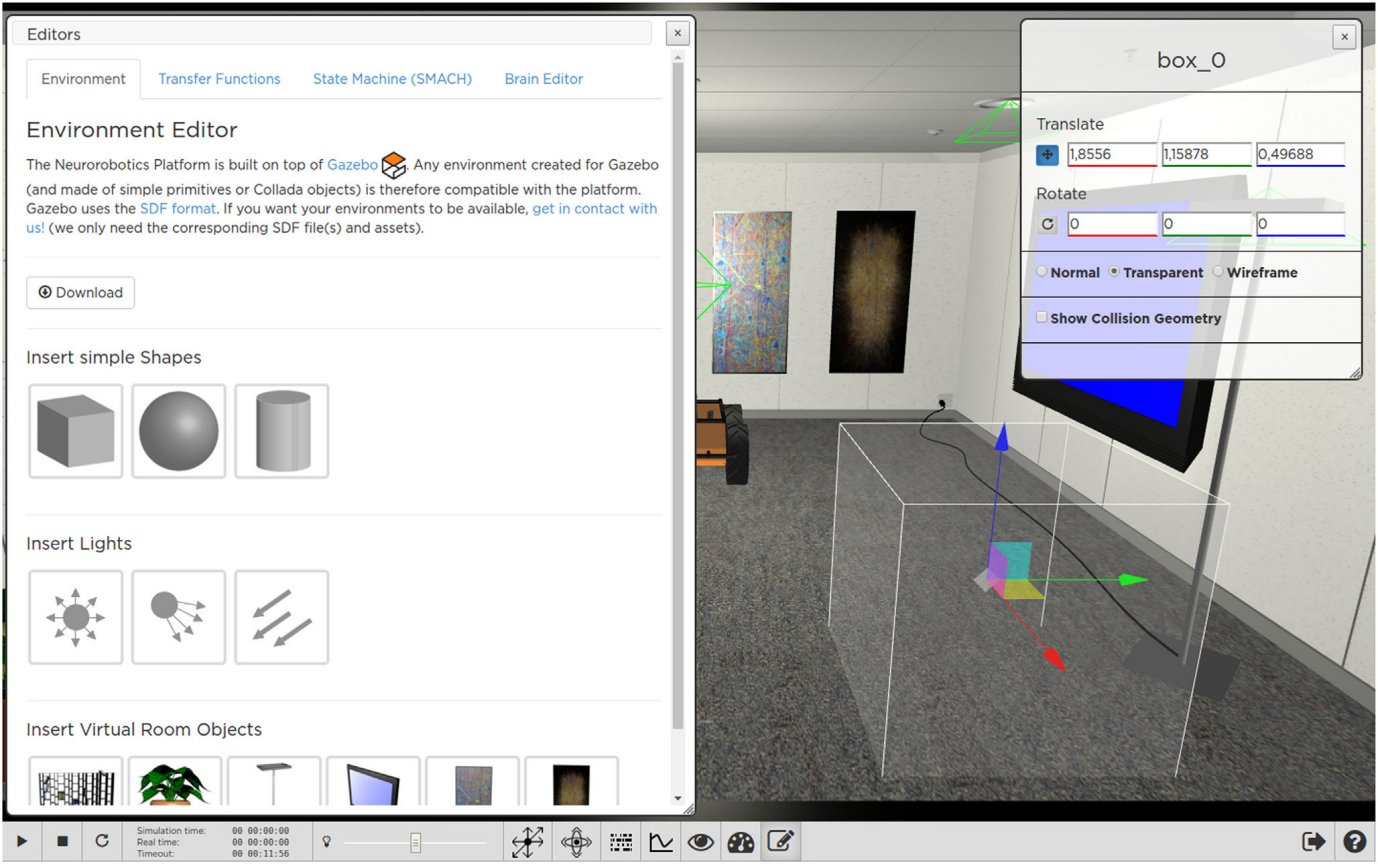
**Adding and editing a new object with the ESV Environment Editor**. The user can change object properties by using appropriate handles or by manually inserting property values (e.g., position coordinates) in a form displayed in a widget.

While running the environment editor, the user is able to move (e.g., translate or rotate) or delete existing objects in the scene, or to place new objects by choosing them from a list of models (Figures 9A and 10).

When the editing of the scene is completed, the user can export the result into the Simulation Description Format (SDF),^4^ either on a local workstation or on the Collab storage of the respective experiment. Once saved, the environment can be loaded at a later time into another different, new or existing, experiment.

Importing a new environment in an existing experiment does not change in any way the workflow of the experiment itself, i.e., it will keep its transfer functions, state machines, the BIBI, and the robot involved.

##### 3.6.3.2 Brain Editor

The Brain Editor (Figure 9D) allows the user to upload and edit custom brain models as PyNN scripts (Davison et al., 2008).

The PyNN script describing the brain model used in the current experiment is shown in a text editor featuring Python keyword and syntax highlighting. It is also possible to define populations (i.e., sets of neurons indices) that can be referred to in transfer functions.

Once the user has finished editing, the new model can be applied without restarting the whole simulation.

##### 3.6.3.3 Transfer Functions Editor

The Transfer Functions (TFs) describe how to transform simulator specific sensory data (such as image, joint angles, forces, etc.) to spiking activity for neural network simulation and vice versa. TFs are defined as Python scripts exploiting the DSL described in Hinkel et al. (2015). Like for the Brain editor, the Transfer Functions Editor (Figure 9B) displays these scripts and enables the user to change them in a text editor pane found in the menu.

From within the editor, the user can create and edit TFs as well as save them to and load them from files. Once edited, the changes can be applied to the simulation. Thus, the user can test immediately the robots’ behavior and, possibly, modify it again resulting in a very short cycle of tuning and testing. Every uploaded transfer function is checked for syntax errors, and several restrictions for Python statements are applied for security reasons.

Furthermore, the user can log TFs’ data to files in the Collaboratory storage to analyze them at a later time. The data format used is the standard Comma Separated Values (CSV). Like for the other editors, the edited TFs can be downloaded on the user’s computer or saved into the Collaboratory storage.

##### 3.6.3.4 Experiment Workflow Editor

The workflow of an experiment is defined in terms of events which are either triggered by simulation time, user interaction, or state of the world simulation. In the current implementation, all events manipulate the simulated environment, as no stimulation of the brain or manipulation of the brain-controlled robot can be performed by the State machine manager.

The workflow is specified in Python code exploiting SMACH (Bohren and Cousins, 2010)—a state machine library integrated into ROS. This approach enables users to specify complex workflows in terms of state machines.

A state machine controlling an experiment interacts with the running simulation by publishing on ROS topics and calling ROS services and can monitor any simulation property published on ROS topics (e.g., simulation time, sensor output, and spiking activity of brain). Like the other editors, the Experiment Workflow Editor (Figure 9C) displays Python scripts and allows the user to change them in a text editor.

### 3.7 Robot Designer

In order to build neurorobotic experiments, the NRP not only has to offer scientists a rich set of robot variants to choose from but also give them the opportunity to integrate virtual counterparts of existing robots in their lab, or robots with the desired morphology for a special, given task. The Robot Designer (RD) hence aims at being a modeling tool for generating geometric, kinematic, and dynamic models that can be used in the simulation environment.

The development from scratch of a custom software (either web or desktop) for modeling and designing a robot is an enormous undertaking, so we decided to adopt existing solutions. In particular, no reasonable web solutions were found, and adapting existing solutions for web would require a considerable effort which would not be counterbalanced by the possible benefits. We chose to use Blender (a powerful and extendable open source software) among the existing modeling softwares, due to its availability for a wide range of platforms with a simple installation process.

Existing extensions for Blender with similar goals were taking into account when developing the Robot Designer. Most notably these are the *Phobos* project^5^ and the *RobotEditor* of the *OpenGrasp* simulation suite (León et al., 2010; Terlemez et al., 2014). The RobotEditor project was finally chosen as the basis of the Robot Designer after an evaluation with competing projects. Afterward, it went through a major refactoring and has been extended by components required for the NRP. These include the aspects of importing and exporting files with support for the Gazebo simulator, additional modeling functionalities, a refined user interface, and data exchange with the Collaboratory storage.

**Figure 11:**
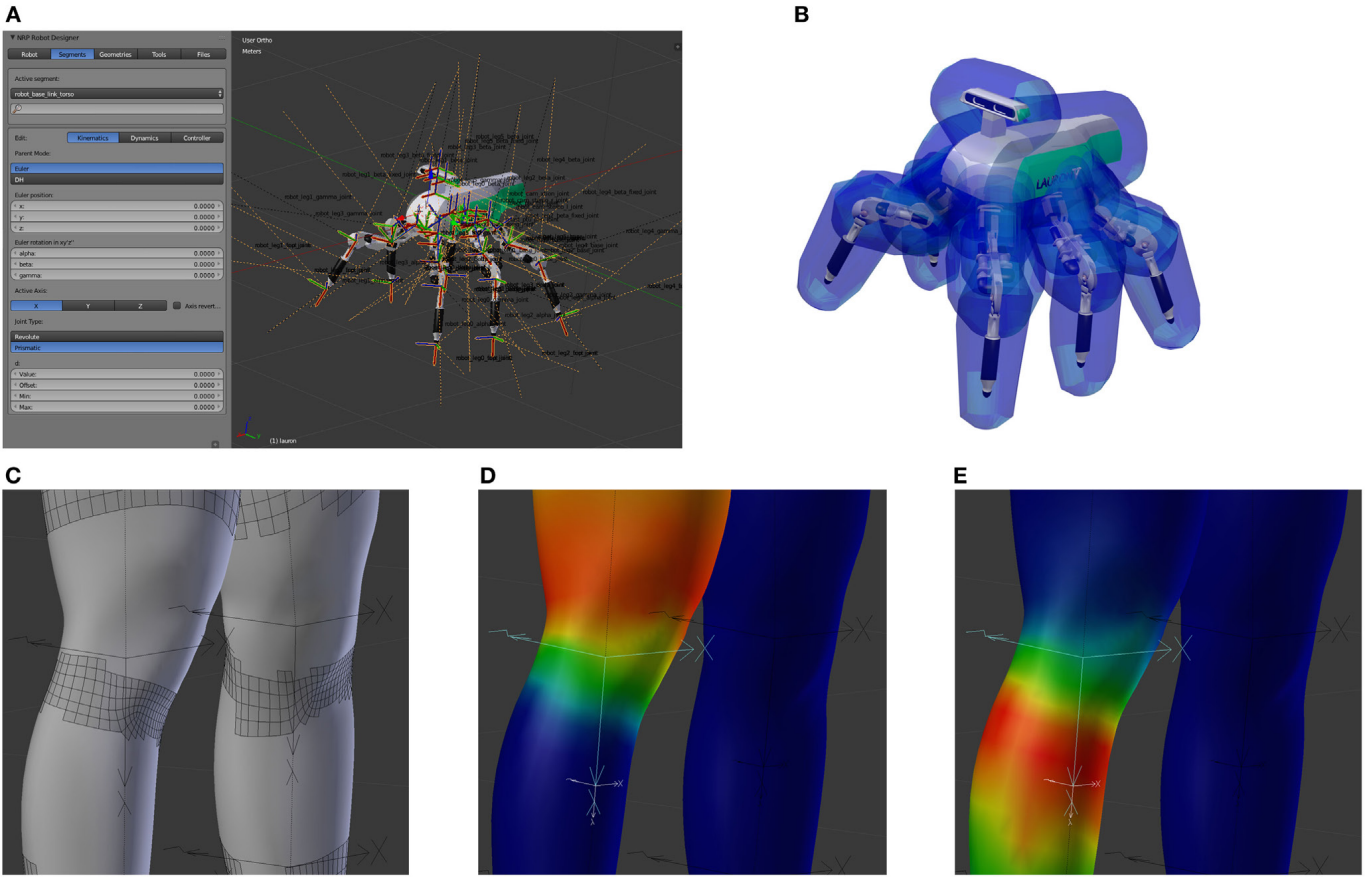
**The Robot Designer**. Using the Robot Designer the user is able to edit kinematic properties of a robot model **(A)**. An example of a completed model of the six-legged walking machine *Lauron V* (Roennau et al., 2014) with a collision model with safety distances is shown in **(B)**. Deformable meshes can be transformed into disjoint rigid bodies for collision model generation **(C)**, by considering the influence of each joint onto the mesh vertexes, e.g., hip joint **(D)** and knee joint **(E)** for a human leg.

The RD provides users with an easy-to-use user interface that allows the construction of robot models and defining kinematic, dynamic, and geometric properties. The robotics-centered user interface of the RobotEditor has been redesigned and allows the user to define kinematic models of robots by specifying segments and joints either using the Euler angles or following the Denavit–Hartenberg convention (Denavit, 1955). The robot dynamic model can be created through mass entities with inertia tensors and controller type with parameters for joints. For geometric modeling, the RD can rely on the vast 3D modeling capabilities provided by Blender, although several additions were made for the automation of robot-related tasks. Figure 11A shows on the left the Robot Designer panel inside Blender while editing the properties of a segment of a six-legged robotic platform, Lauron V (Roennau et al., 2014). The plugin provides overlays for the 3D view that shows reference frames and names for each robot joint, thus facilitating editing.

The original code of the RobotEditor has been heavily refactored, and the documentation for users and developers of the robot designer and the core framework has been greatly expanded. The core framework offers many additional features such as resource handling, logging to external files, debug messages with call stacks, and the concept of pre- and postconditions checking for validation of functionality.

Data exchange with the NRP and with ROS has been a major aspect of the development of the Robot Designer. For this reason, support for the widespread *Unified Robot Description Format (URDF)*^6^ file format has been added and been improved in several ways during the development. An *XML schema definition* file has been generated for this file format which then made it easier to generate language-specific bindings^7^ requiring only a small interface between internal data types and the representation in the XML document (see Section 3.3).

In addition to exporting raw URDF files, the Robot Designer also supports novel features unique to the robot simulation of the NRP. Above all, this includes generating input to a Gazebo plugin loaded by the CLE. It automatically generates software implementing necessary joint controllers for position and/or velocity control. This additional information is not included in the URDF standard and is stored together with the model in the same file. For the user of the NRP, this means that different controller types and parameters for each joint can conveniently be specified directly in the designer and become available in the simulation without the need of writing additional joint controller software and its deployment on the platform servers. The persistent storage and data exchange mechanisms of the Robot Designer offer the user the option to encapsulate models into installable and zipped ROS packages.

Several modeling and automatization features were also added to the already feature-rich modeling software. Collision models can automatically be created from the geometric model of a robot either by computation of its complete convex hull or an approximate convex hull with a fixed polygon count and an additional safety distance to the original mesh (see Figure 11).

When generating collision models for deformable geometries, the underlying mesh, where each vertex has a linear influence of multiple joints, has to be transformed into several disjoint rigid bodies. The RD can perform this transformation based on several rules (see Figures 11C–E) as it is demonstrated on the showcase of a mesh created with the *MakeHuman*^8^ project.

Finally, automatic robot generation from the mathematical kinematic model has been added as an experimental feature.

The Robot Designer provides an easy installation process. To download and activate the software, an installation script that runs within Blender has to be executed by the user. This script, the Robot Designer itself, and its documentation are hosted on a publicly available repository.^9^

## 4 Software Development Methodology

The NRP is developed within the Scrum agile software development framework (Schwaber and Beedle, 2002). The basic unit of development, in Scrum parlance, is called a *Sprint*. It is a timeboxed effort, which is restricted to a specific duration of either 2 or 3 weeks. This methodology provides a reactive environment, able to deal with changing requirements and architectural specifications.

The Scrum process includes daily stand-up meetings, where each team of developers discusses about how they are working to meet the sprint goals. At the end of the sprint, a review meeting is held; the whole NRP team is present, and the members make demonstrations of the software in stable development status. Each completed task provides a new feature to the user, without breaking compatibility with the current code base. Thus, at the end of each sprint, there is a new shippable platform that provides new features.

The NRP software process uses industry standards for quality control. The acceptance criteria of the version control system include the necessity of a code review by, at least, a second programmer, while a continuous integration system ensures that new code does not introduce regressions by executing a set of unit tests. Moreover, code coverage criteria ensure that at least 80% of the code is covered during tests and coding standards are enforced by automatic static code analysis tools (PEP8 and Pylint). Each build in the continuous integration system also produces the software documentation documenting the APIs and comprising software usage examples. A summary of quality control statistics regarding the main NRP repositories is presented in Table 1. No repository has PEP8 or Pylint errors.

**Table 1:** **Summary of quality control statistics for the NRP repositories**.

Repository	Total lines	Tests	Line coverage (%)	Branch coverage (%)
CLE	2,944	147	88	100
Backend	3,045	239	93	100
Frontend (ESV)	2,427	455	95	87
Experiment control	455	46	96	100

## 5 Use Cases for the Neurorobotics Platform

In order to assess the functionalities of the NRP, several experiments were designed. These experiments, albeit simple in nature, aim at demonstrating various features of the platform. The first use case is just a proof of concept: a very simple brain model is connected to a robot via TFs in order to have a complete action–perception loop performing a Braitenberg vehicle experiment (Braitenberg, 1986). Results show that the two simulations are properly synchronized and the experiment is correctly performed.

Then, an experiment that makes use of the TF framework capability of implementing classic robotic controllers was designed and implemented. In this case, the robot–brain loop is short-circuited, and a controller implemented inside a TF is used to perform sensorimotor learning with a robotic arm.

Finally, in order to demonstrate the extensibility of the framework, an already existing computational model of the retina was integrated inside the platform and used to perform bioinspired image processing.

### 5.1 Basic Proof of Concept: Braitenberg Vehicle

This experiment was designed in order to validate the overall functionalities of the NRP framework. By taking inspiration from Braitenberg vehicles, we created an experiment where a four-wheeled robot equipped with a camera (Husky robot from Clearpath Robotics) was placed inside an environment with two virtual screens. The screens can display a red or blue image, and the user can interact with them by changing their displayed image by using the ESV. The robot behavior is to turn counterclockwisely until it recognizes the red color and then to move toward the screen displaying the red image.

**Figure 12:**
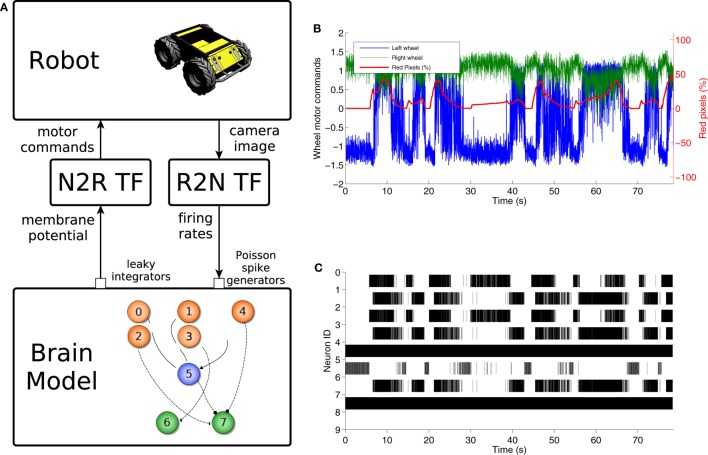
**Braitenberg vehicle experiment**. The control model **(A)** uses a color detection robot to neuron transfer function to convert the image into spike rates, a simple spiking neural network comprising 8 neurons and a neuron to robot transfer function that translates membrane potentials into motor commands. The motor signals sent to the robot wheels are directly correlated with the red pixels’ percentage in the camera image **(B)**. This is also reflected by the changes in brain activity during the trial **(C)**.

The overall control architecture can be observed in Figure 12A. Identification of the red color is done in a robot to neuron transfer function where the image coming from the robot camera is processed via a standard image processing library, OpenCV, in order to find the percentage of red pixels in the left and right halves of the image. Such information is then translated into firing rates and sent as an input to *Poisson spike generator* devices. These devices provide the input for a simple Brain Model comprising 8 neurons. Among these, three are sensor neurons, receiving inputs from the spike generator devices, and two are actor neurons, encoding the generated motor commands. The behavior of the neural network is to make one of the two actor neurons have a much higher firing rate compared to the other if no input encoding red pixels is present, while making the two neurons fire with a firing rate that is more similar the more red is present in the two image halves. Two *leaky integrator* devices receive input from the actor neurons and are used in the neuron to robot transfer function responsible for the generation of motor commands. In particular, membrane potential of these devices is used to generate motor commands for the left and right wheels such that when the two actor neurons’ firing rates differ, the wheels turn in opposite directions, effectively turning the robot, and when the firing rates match, the wheels move in the same direction, moving the robot forward.

The behavior of the experiment is shown in Figures 12B,C, where it can be observed that every time there is a rise in the red percentage on the image there is an increase in the spike rate of neuron 7 so that it matches that of neuron 8. Then, the generated motor commands change accordingly, and the wheels move in the same direction, effectively moving the robot forward.

### 5.2 Classic Robot Controller: Sensorimotor Learning

**Figure 13:**
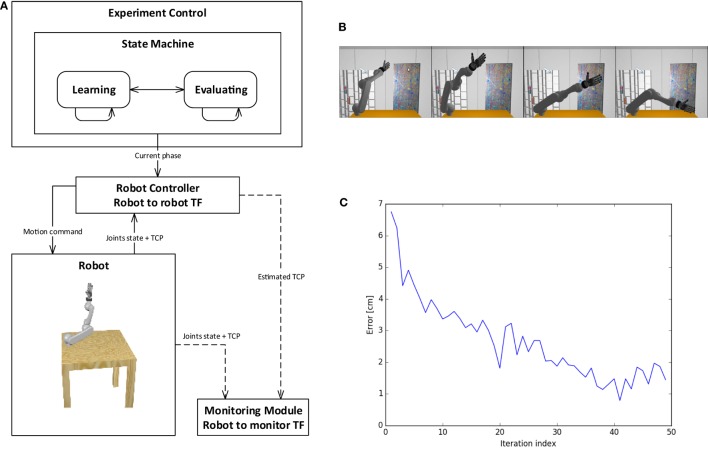
**Sensorimotor learning experiment**. The control schema includes a state machine for experiment control using a classical robot controller and monitoring module **(A)**. The state machine switches between the two phases of the experiment **(B)**: motor babbling phase for training, then evaluation of TCP prediction. After each training iteration, the prediction error decreases, reaching 1 cm of accuracy for TCP estimation after 40 iterations **(C)**.

The goal of the experiment is to learn sensorimotor coordination for target reaching tasks to be used in future manipulation experiments. In particular, the experiment aims at predicting a forward model for an anthropomorphic arm, by estimating the tool center point (TCP) position from the current joint configuration. In its current form, the experiment consists of two phases, repeated every iteration: in the first phase, shown in Figure 13B, the robot explores the working space and learns its kinematics by performing random movements (i.e., motor babbling), observing its TCP position and corresponding joint configuration; in a second phase, the model is evaluated by moving the arm in a random position and comparing the TCP predicted by the learnt kinematic model with the real one. This experiment does not use any brain model, thus it shows that the NRP also provides a framework for implementing classic robot controllers.

The control schema of the experiment is presented in Figure 13A. The state machine for experiment control switches between the different phases and communicates the current phase to the robot controller. The robot controller implements a supervised learning method, the Kinematic Bezier Maps (KBM) (Ulbrich et al., 2012), and communicates directly to the simulated robot in a robot to robot transfer function. During the learning phase of each iteration, the robot controller moves the arm in a random joint configuration and feeds this information, alongside the real TCP of the attached end effector, to the KBM model. During the evaluation phase, the arm is moved into another random joint configuration, and the KBM model is used to predict the position of the new TCP. This information is sent to the monitoring module. The monitoring module also gathers information from the simulation, such as the real TCP and joint values. This information can be stored, displayed, or further processed. In particular, this information is used to compute the accuracy of the KBM prediction. Figure 13C shows the learning curve for the training of the kinematic model, where the error is computed as the distance in space between the predicted TCP and the real one. It can be noticed that the error decreases during training iterations, reaching an accuracy of 1 cm.

### 5.3 Integration of Bioinspired Models: Retinal Vision

In order to have full biologically inspired closed loop controllers, the transfer functions should also make use of neuroscientific models of sensor information processing from one side and motion generation on the other. As a first step in this direction, a model of the retina was included in the NRP as a robot to neuron transfer function (Ambrosano et al., 2016).

The model chosen for the integration was *COREM*, a computational framework for realistic retina modeling (Martínez-Cañada et al., 2015, 2016), that provides a general framework capable of simulating customizable retinal models. In particular, the simulator provides a set of computational retinal microcircuits that can be used as basic building blocks for the modeling of different retina functions: one spatial processing module (a space-variant Gaussian filter), two temporal modules (a low-pass temporal filter and a single-compartment model), a configurable time-independent non-linearity, and a Short-Term Plasticity (STP) function.

The integration work proceeded by creating Python bindings for the C++ COREM implementation and by adding the appropriate functions that could feed the camera image in the model and extract the retinal output without changing the core implementation. Such implementation provides, as an output, analog values representing the intensity of presynaptic currents of ganglion cells (Martínez-Cañada et al., 2016). Thus, the retina simulator now provides an interface that is callable by the transfer function framework. Moreover, the retina model is defined via a Python script, which can be uploaded by the user.

**Figure 14:**
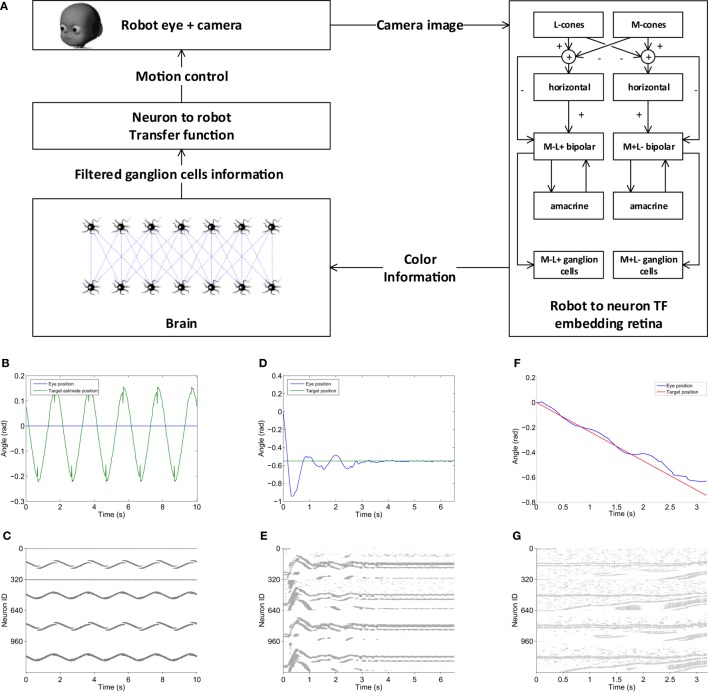
**Visual tracking with retinal image processing experiment**. **(A)** The visual tracking model embeds a retina model capable of exploiting red–green opponency as a robot to neuron transfer function, a two-layer brain model that filters color information and a neuron to robot transfer function that uses the filtered target position information to generate motor commands for the eye. The model is able to correctly detect a moving target as it shown in panels **(B,C)**, where the target estimated position and corresponding brain activity are presented. When the eye moves, a more noisy retinal input is produced, but the brain model is still able to filter it and performing step response tasks **(D,E)** and pursuit of linearly moving targets **(F,G)**.

In order to test the proper integration of the retina simulator, a first experiment that involves visual tracking of a moving target via a retinal motion recognition was designed. The environment setup consisted of placing the simulated robot (iCub humanoid robot) in front of a virtual screen. The screen displayed a red background with a green circle that can be controlled (target). The overall control scheme can be observed in Figure 14A. This model improves a previously designed visual tracking controller implemented using the same Brain Model of the experiment described in Section 5.1 (Vannucci et al., 2015). A model of retinal *red–green opponency* was used as a robot to neuron transfer function. This opponency is a basic mechanism through which color information is transmitted from the photoreceptors to the visual cortex (Dacey and Packer, 2003). This model has two retinal pathways whose outputs are more sensitive to green objects appearing in receptive fields that were earlier stimulated by red objects and vice versa. Only one horizontal stripe of the retinal output, intersecting the target position, is extracted and fed into a brain model, via current generator devices. The brain model consists of 1,280 integrate and fire neurons organized in two layers. The first layer acts as a current to spike converter for the retina ganglion cells, while in the second layer, every neuron gathers information from 7 neurons on the first layer, acting as a local spike integrator. Thus the second layer population encodes the position of the edges of the target in the horizontal stripes (corresponding to 320 pixels). Such information, encoded as a spike count, is then used by the robot to neuron transfer function in order to find the centroid of the target. Information about the target centroid can also be used to generate motor commands that make the robot perform visual tracking of the moving target.

The accuracy of target detection can be observed in Figure 14B, where the results of a trial where the target was moved with a sinusoidal motion and the robot eye was kept still are shown. It can be noticed that the target motion is fully captured by the model and this is reflected in the corresponding brain activity (Figure 14C). Figure 14D shows the behavior of the controller during a step response toward a static target: the eye is able to reach the target, albeit with some overshooting. Comparing the brain activity during this task (Figure 14E) with the target detection one (Figure 14C), it is noticeable how the retinal output is noisier during this trial. This is due to the intrinsic motion detection capabilities of the retina as the activity of ganglion cells increases when some motion is detected. Nevertheless, the second layer of neurons in the brain model (lower half) is still able to filter out activity of the first layer (upper half) not relative to the target, thus its position can be computed with more accuracy. Similarly, during a task where the robot had to follow a target moving linearly, the eye motion produces some noise in the retinal output (Figure 14G), but the controller is still able to extract the target position and successfully perform the task (Figure 14F).

## 6 Future Developments

The features detailed in the previous sections describe the first release of the Neurorobotic Platform. The development of the platform will continue, in order to provide even more simulation capabilities and features to the end user.

Short-term development plans include integration with the Brain Simulation Platform and the Neuromorphic Computing Platform, as described in Section 3, as well as an extension of the CLE that will be able to orchestrate distributed brain simulations, giving it the potential to simulate larger brain models in shorter times, that will lead to the integration with the High Performance and Data Analytics Platform.

The State machines manager will be extended in order to respond also to event produced by the robot behavior, such as the robot entering a certain area of the environment or performing an action, allowing the user to design more complex experiments. The user will also be able to design the experiment workflow using a graphical support included in the ESV GUI, with a timeline-based view that allows users to directly select objects and properties in the 3D environment and create events based on their state in the world simulation. Moreover, we plan to support fully automated repetitions of experiments including success evaluation for each trial.

Finally, the users will be able to upload environment built offline from custom physicals models within the platform, greatly enhancing the environment building capabilities. At the same time, the Robot Designer will be extended to include support of external debuggers, static type checking, and code analysis. It is also planned to separate the core framework from the Robot Designer and release it as an independent project to facilitate plug-in development in Blender in general.

## 7 Conclusion

This paper presented the first release of the HBP Neurorobotics Platform, developed within the EU Flagship Human Brain Project. The NRP provides scientists for the first time with an integrated toolchain for *in silico* experimentation in neurorobotics, that is, to simulate robots with neuro-controllers in complex environments. In particular, the NRP allows researchers to design simulated robot bodies, connect these bodies to brain models, embed the bodies in rich simulated environments, and calibrate the brain models to match the specific characteristics of the robots sensors and actuators. The resulting setups can permit to replicate classical animal and human experiments *in silico* and ultimately to perform experiments that would not be possible in a laboratory environment. The web-based user interface allows to avoid software installation and the integration within the HBP collaboratory portal gives access to storage and computing resources of the HBP. Users can run experiments alone or in team, and this can foster collaborative research allowing the sharing of models and experiments.

In order to demonstrate the functionalities of the platform, we performed three experiments, a Braitenberg task implemented on a mobile robot, a sensory-motor task based on a robotic controller, and a visual tracking embedding a retina model implemented on the iCub humanoid robot. These use cases make it possible to assess the applicability of the NRP in robotic tasks as well as in neuroscientific experiments.

The final goal of the NRP is to couple robots to detailed models of the brain, which will be developed in the HBP framework. It will be possible for robotics and neuroscience researchers to test state of the art brain models in their research. At the current stage, the results achieved with the NRP demonstrate that it is possible to connect simulations of simple spiking neural networks with simulated robots. Future work will focus on the integration of the mentioned neural models. In addition to this, the integration of high-performance computing clusters and neuromorphic hardware will also be pursued in order to improve execution time of spiking neural networks replicating detailed brain models. All informations relative to the NRP, including how to access it and where to find the code, are available on the plaftform website: http://neurorobotics.net.

## Author Contributions

All authors listed have made substantial, direct, and intellectual contribution to the work; they have also approved it for publication. In particular, EF, LV, AlAm, UA, SU, CL, AK, and M-OG contributed to the design of this work; EF, LV, AlAm, UA, SU, JT, GH, JK, IP, OD, NC, and M-OG contributed to the writing of the manuscript; ER and PM-C designed the retina model, implemented it in the COREM framework, and collaborated in integrating it into the NRP, together with LV, AlAm, and JK; GK, FR, PS, RD, PL, CL, AK, and M-OG contributed to the conception and design of the NRP; and EF, LV, AlAm, UA, SU, JT, GH, JK, IP, PM, MH, AR, DaPl, SD, SW, OD, NC, MK, AR, AxvoAr, LG, and DaPe developed the NRP.

## Conflict of Interest Statement

The authors declare that the research was conducted in the absence of any commercial or financial relationships that could be construed as a potential conflict of interest.

## Footnotes

^2^https://www.blender.org/.

^3^https://www.angularjs.org/.

^4^http://sdformat.org/.

^5^https://github.com/rock-simulation/phobos.

^6^http://wiki.ros.org/urdf/XML.

^7^https://pypi.python.org/pypi/PyXB.

^8^http://www.makehuman.org/.

^9^https://github.com/HBPNeurorobotics/BlenderRobotDesigner.

## APPENDIX

### A Functional Requirements

As stated in Section 2.1, the itemized functional requirements should be interpreted as implemented platform features. All the listed requirements are to be satisfied by the NRP as a whole. For the sake of clarity, the requirements are grouped by topic.

#### A.1 Design and Editing

##### A.1.1 Robot

- Assemble a virtual robot
  – shall enable the user to load a ready-made robot from a graphical library
  – shall enable the user to assemble a robot using ready-made robot parts from a graphical library
  – shall provide standard sensors and actuators to the ready-made or block-assembled robot
- Define a kinematic chain
  – shall enable the user to define a kinematic chain by building a tree-like structure of the single links
  – shall enable the user to group kinematic chains
- Robot editing
  – shall enable the user to select parts of the robot
  – shall enable the user to edit a robot parts graphical attributes or physical attribute
- Save/load designed robot
  – shall enable the user to save a model of a virtual robot
  – shall provide well-defined file formats (e.g., XML, URDF, and SDF)

##### A.1.2 Environment

- Assemble and model virtual environment
  – shall enable the user to instantiate any number of objects
  – shall enable the user to remove objects from the environment
  – shall enable the user to interactively change objects poses and orientations
  – shall provide a GUI to view the object parameters
  – shall provide a GUI to edit the object parameters
  – shall enable the user to select (e.g., drag and drop) objects from a local library of available objects
- Load/save virtual environment
  – shall provide a custom environment which can be loaded in a new experiment
  – shall provide a custom environment which can be loaded in an existing experiment
  – shall enable the user to save the environment status at any moment during the simulation/editing
  – shall provide the user to store the environment in a well-defined file format

##### A.1.3 Brain

- Create Brain Model
  – shall support binary format for data-driven brain model representation
- Save/load and edit brain models
  – shall enable the user to save brain models
  – shall enable the user to reload brain models
  – shall provide a visual interface to edit brain models

##### A.1.4 Brain–Body Interface

- Transfer Modules
  – shall provide input or output variables that are produced or consumed by the brain simulation (currents, spikes, and spike rates)
  – shall provide input or output variables that are produced or consumed by the sensor or the actuators of the robot
  – shall provide data from both simulators that can be consumed by monitoring or debugging interfaces
  – shall produce suitable output for experiment data gathering, saving it to a common file format
  – shall handle intensive computations such as the simulation of a spinal cord or retina model
  – shall hold a state, defined as a set of variables that keeps some values in between the loops
- Transfer modules editing
  – shall enable the user to save and reload transfer modules
  – shall enable the user to edit transfer modules through a visual interface
  – shall enable the user to select populations of neurons
  – shall enable the user to label populations of neurons
  – shall enable the user to connect groups of neurons to a transfer module and vice versa
  – shall enable the user to connect sensors and actuators to a transfer module and vice versa

##### A.1.5 Experiment

- Configuring and loading/saving an experiment setup
  – shall enable the user to select the virtual environment
  – shall enable the user to select the Neurobot to use in the experiment
  – shall provide user to load/save a definition of an experiment setup
- Defining action sequences
  – shall enable the user to define events (e.g., change of light intensity and moving of an object) occurring at a certain point in time
  – shall enable the user to define the properties of an event (e.g., duration)
  – shall enable the user to specify complex events (by combining single events)
  – shall enable developers to define more complex actions a scripting-interface

#### A.2 Simulation

##### A.2.1 Simulation Consistency and Synchronization Mechanisms

- Simulation control
  – shall support start, stop and pause of an experiment at any time
  – shall reset every component of the simulation at any time
  – shall be capable of exposing its internal simulation execution speed
  – shall be presented to the user in a visual interface
  – shall provide an interface to modify any variable parameters to control the details of the simulation
  – shall be completely reproducible
- Physics simulation
  – shall maintain a consistent model of the world and the robot during the execution of an experiment
  – shall maintain a world clock to update the world state in suitable time-slices
- Synchronization
  – the loop between the brain simulator and the WSE shall operate at a rate of an order of magnitude of 0.1 ms of simulated time
  – the world clock shall be synchronized to the brain simulation clock to achieve a consistent, overarching notion of time
  – the simulation framework shall be capable of injecting actions into the simulation of the virtual world as defined by the experimenter

##### A.2.2 Interactive Visualization

- Simulation control/editing
  – shall enable the user to control the simulation through the GUI
  – shall enable the user to live edit the experiment configuration using the GUI
- Simulation monitoring
  – shall display a 3D rendering of the world scene
  – shall enable the user to navigate the 3D scene
  – shall display measurements from the two simulations
  – shall enable multiple users to view the same running simulation at the same time
